# Practical application of SAM for breast nodules segmentation

**DOI:** 10.3389/fonc.2026.1756011

**Published:** 2026-03-10

**Authors:** Wei Fan, Ansheng Li, Mingze Xu, Wei Sun, Fengyuan Man

**Affiliations:** 1 Department of Radiology, Rocket Force Characteristic Medical Center of the Chinese People’s Liberation Army, Beijing, China; 2 Department of MRI Diagnosis, The Second Affiliated Hospital of Harbin Medical University, Harbin, China; 3 Department of Interventional Therapy, National Cancer Center/National Clinical Research Center for Cancer/Cancer Hospital, Chinese Academy of Medical Sciences and Peking Union Medical College, Beijing, China

**Keywords:** breast cancer, breast nodules segmentation, DCE, magnetic resonance imaging, SAM

## Abstract

Breast cancer is one of the most common and deadly diseases that threaten women’s health worldwide, and early and accurate breast nodule segmentation is of great significance for the early detection, diagnosis and treatment of breast cancer. However, due to the limitation of medical annotated data, the training segmentation models for medical images is still challenging. The Segment Anything Model (SAM) is a foundational model that interactively segments target objects. Although significant achievements have been made in natural images, there are still challenges in the application in medical images. In this paper, the effect of SAM on breast nodule segmentation was studied from three aspects: initial weight, organ (breast) mask and prompt box, so as to explore the feasibility of breast nodule segmentation. Through a series of experiments on the data collected in this paper, it is found that the use of MedSAM initial weights and the use of single individual fixed prompt boxes can obtain better segmentation results, and can take into account practical application problems.

## 1 Introduction

Breast cancer is one of the most common and fatal diseases in women, with about 2.3 million women diagnosed with breast cancer in 2020 and 685, 000 of them dying as a result (1). Early diagnosis and treatment significantly reduce the mortality rate of breast cancer patients. Biopsy can cause damage and discomfort to the patient’s body, while magnetic resonance imaging (MRI) is a non-invasive examination with high repeatability. MRI has the advantages of non-ionizing radiation and high soft tissue resolution, which has high application value in the differentiation and diagnosis of breast cancer.

Breast nodule segmentation is a technique that separates the target area and background information in a patient’s medical image (2, 3). There are two main types of segmentation methods for breast nodules in MRI: traditional segmentation methods and segmentation methods based on deep learning. Traditional segmentation methods can be divided into supervised and unsupervised methods, among which edge detection, region growth, threshold segmentation, and clustering (4–7) (K-means, fuzzy c-means) belong to unsupervised methods, and classification-based models (8) (SVM, KNN, random forest, etc.) and regression-based models (9, 10) belong to supervised methods.

The deep learning method applied in the field of medical image segmentation mainly uses the encoder-decoder structure (11–13), in which the image to be processed is input into the encoder to obtain a feature map, and then the feature maps is decoded by the decoder to obtain the target object. The encoder-decoder structure has been widely used in medical image processing research based on deep learning, the most representative of which are Full Convolutional Network (FCN) (14) and U-Net (15). Due to the limitations of the model architecture, convolutional neural networks and recurrent neural networks cannot fully integrate information from long distances, which leads to certain limitations in dealing with long-range dependencies. In 2017, Ashish proposed a Transformer network framework using attention mechanisms (16). Transformer is a deep learning model based on attention mechanism, which was originally proposed for natural language processing tasks. The self-attention (SA) mechanism is able to extract the appropriate contextual information for each position. Capture long-range dependencies to improve global understanding of the task.

However, developing and training segmentation models for new medical imaging data and/or tasks is practically challenging, due to the expensive and time-consuming nature of collecting and curating medical images. These difficulties could be significantly mitigated with the advent of foundation models (17) and zero-shot learning (18). Foundation models are neural networks trained on an extensive amount of data, using creative learning and prompting objectives that typically do not require traditional supervised training labels, both of which contribute towards the ability to perform zero-shot learning on completely new data in a variety of settings. Foundation models have shown paradigm-shifting abilities in the domain of natural language processing (19).

In 2023, Meta AI proposed a “segment anything model” (SAM) model built on the Transformer framework (20), which demonstrated strong performance in image segmentation tasks. The SAM model uses 110.01 million high-resolution images and 1.1 billion high-quality segmentation labels. SAM is designed to implement segmentation tasks in general, rather than interactive segmentation with high confidence. SAM performs well in general, but it misses subtle structures, while medical image segmentation is a highly vertical, domain-specific task that requires attention to tiny lesions. Previous studies combined various segmentation models with mammography images (21, 22). Studies have shown that the segmentation performance of SAM models on medical images is not satisfactory (23–25). Recent studies have increasingly focused on applying the Segment Anything Model (SAM) to medical imaging, with multiple works demonstrating encouraging performance across a variety of segmentation tasks (26–28). In some studies, a pre-trained model provided by Meta AI was used as the base model to fine-tune medical image data, and the resulting model in the dataset was significantly improved compared to the SAM model (29). In the field of breast imaging, SAM-based methods have primarily focused on ultrasound images, while studies applying SAM and its variants to MRI segmentation have remained limited, which motivated the present study (30–32).

The purpose of this study was to explore the practical effects of various factors on the segmentation results of breast nodules using the SAM model, so as to improve the practicability of SAM breast nodule segmentation. The effects of initial weight, breast mask and segmentation prompt box on the segmentation performance of the model were mainly studied, and the feasibility of SAM to complete the segmentation of breast nodules was discussed.

## 2 Materials and methods

### 2.1 Data

A total of 103 female breast MRI data were collected, including 80 cases of malignant nodules and 23 cases of benign nodules. The imaging sequence was DCE sequence as follows: the field of view was 286×286 mm² to 320×320mm², the pixel spacing was 1.022×1.022 mm² to 1.156×1.156 mm², and the imaging layers were 30 to 160 layers. This study was a retrospective study and was approved by a local ethic committee in Rocket Force Characteristic Medical Center of the Chinese People’s Liberation Army.

The contours of the nodules were outlined by an experienced radiologist as the ground truth (GT) for segmentation. At the same time, the segmentation of the mammary region was performed using the U-net network, and the radiologist adjusted it to form a breast mask (the organ mask).

The mainstream evaluation criteria for benign and malignant breast nodules are the BI-RADS (Breast Imaging Reporting and Data System) classification developed by the American College of Radiology, which mainly distinguishes breast nodules from morphology, aspect ratio, etc. The volume of benign nodules in this experimental data was 4485.16 ± 6630.11mm^3^, and the volume of malignant nodules was 15824.30 ± 20216.09mm^3^.

### 2.2 Image preprocessing

The images were separated the left and right breast, and only the breast with nodules was selected as the data processing target, and after this operation, the data size became 256×256×40.According to the histogram of the gray value of the original image, the pixels (cases) with gray values less than 0.5% and greater than 99.5% of the overall gray scale were eliminated, and then the gray scale of the selected pixels was linearly adjusted to the range of 0-255.The small area of the connected domain was removed on each slice, the threshold of the connected domain was set to 50.

SAM was designed for a three-channel RGB image, the processed slice data was copied three times as a three-channel data. Finally, the image data was resized to 1024×1024×3, and the gray value were normalized to the range of 0-1. The ground truth was also resized to 1024×1024, and the breast mask remained 256×256. For the input data multiplied with the mammary mask (hard-attention), the slice (256×256) was multiplied by the mammary mask, and then resized to 1024×1024×3. The preprocessing pipelines for the training, validation, and test sets were methodologically identical ensuring that no evaluation bias was introduced during preprocessing.

### 2.3 Variation in SAM

**Figure 1:**
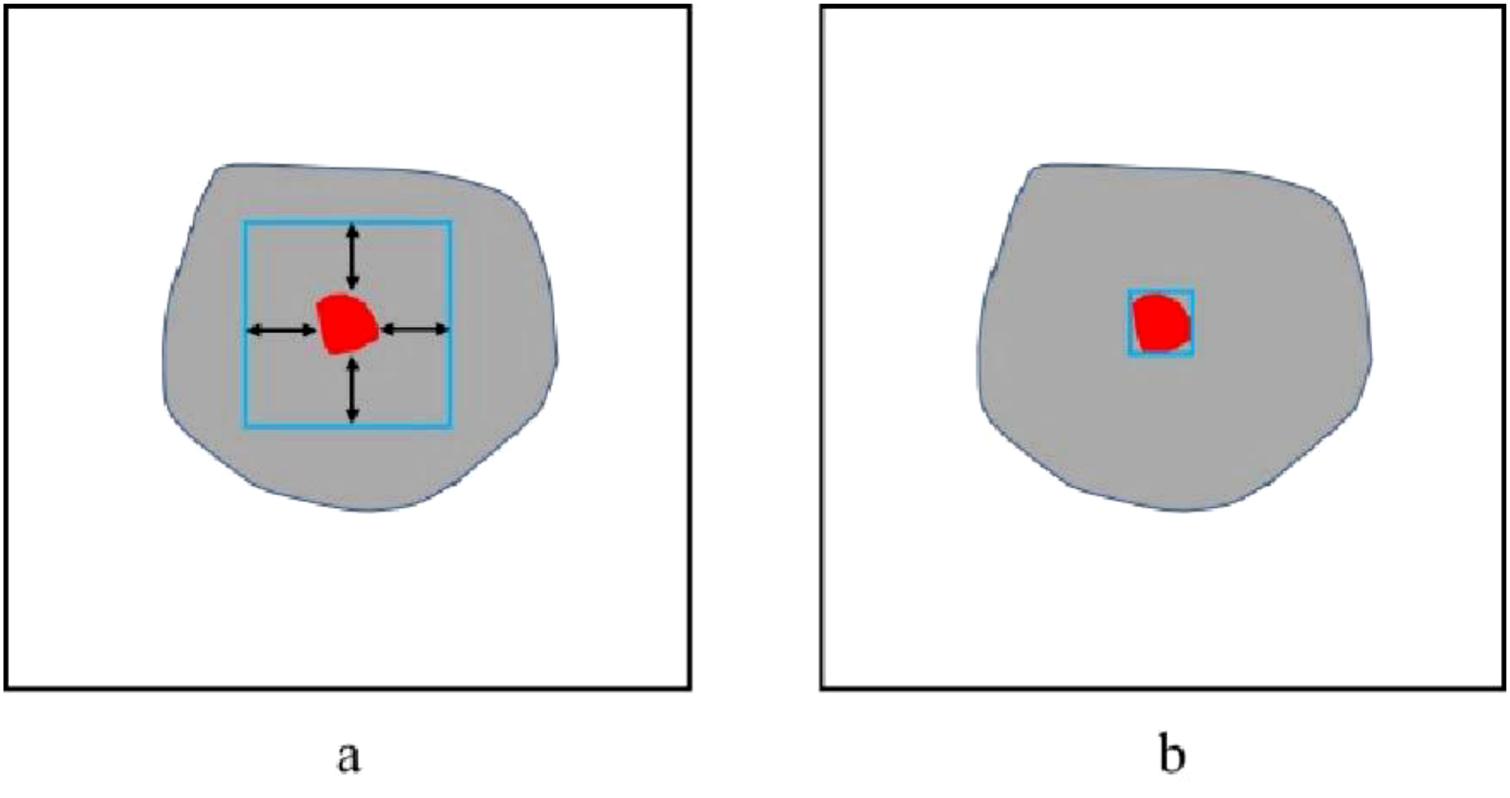
Schematic diagram of the prompt box. **(a)** Fixed box, **(b)** Adaptive box.

There were two types of prompt box, the adaptive prompt box and the fixed prompt box. An adaptive prompt box was constructed according to ground truth of each 2D nodule, and the prompt box was a rectangle randomly expanded by 1–20 pixels with the bounding box of the gold standard of the nodule. The fixed prompt box was constructed on the slice with the maximum nodule areas. The bounding box of the nodule area were expanded 100 pixels to form the fixed prompt box, and for the same case the prompt boxes of each slice were the same. The adaptive prompt box and fixed prompt box were showed in **Figure 1**.

The organ mask used in this paper was the breast mask. There were three way using organ mask: multiplying the input image with the mask before entering the SAM model (hard mask), being as the mask of SAM model (soft mask), or None-mask.

The initial parameters were from the well-trained SAM (8) or MedSAM (24). The corresponding variation of SAM was listed in **Table 1**.

**Table 1:** Relationship between different experimental codes and variables (• represents used, ○ represents unused).

Model	Initial weights		Box		Mask		
	SAM	MedSAM	fixed	Adaptive	Hard Mask	Soft mask	No mask
SAM	•	○	•	○	○	•	○
MedSAM-m	○	•	•	○	•	○	○
MedSAM-a	○	•	○	•	○	•	○
MedSAM-nm	○	•	•	○	○	○	•
SAM-nm	•	○	•	○	○	○	•
MedSAM-anm	○	•		•	○	○	•
MedSAM	○	•	•	○	○	•	○

### 2.4 Models building

Because of the elimination of the small connected domain in the pre-processing stage, four benign samples with too small nodules were rejected. The model was trained and tested by a 5-fold cross-validation on the remaining data (99 cases), and each fold was independent of the training and test data.

The loss function takes the weighted sum of the cross entropy loss (CE) and the dice loss as follows Equations 1–3:

(1) $$\begin{array}{c} L=L_{CE}+L_{Dice} \end{array}$$

(2) $$\begin{array}{c} L_{CE}=-\frac{1}{N}\sum_{i=1}^{N}g_{i}\log s_{i} \end{array}$$

(3) $$\begin{array}{c} L_{dice}=1-\frac{2\sum_{i=1}^{N}g_{i}s_{i}}{\sum_{i=1}^{N}g_{i}^{2}+\sum_{i=1}^{N}s_{i}^{2}} \end{array}$$

where *s_i_, g_i_* denote the voxels in the segmentation result and the ground truth, respectively, and *N* denote the overall voxels number of each sample.

The network was optimized by AdamW optimizer (β_1_ = 0.9, β_2_ = 0.999), and the initial learning rate was 0.0001 and weight decay was 0.01. The batch size was 2 and no data augmentation was used, and the training epoch was 100. The training environment of the model was Nvidia Tesla V100 (32G) GPU.

The convolution-based classic U-Net and Transformer-based U-Net(Unetr), as two representative models, were compared with various variants of SAM. To ensure methodological rigor and equitable comparison all baseline models (U-Net and Unetr) were trained under identical settings consistent with the SAM-based models: 1) uniform preprocessing pipelines were enforced across all experimental conditions, with identical intensity normalization and resampling procedures; 2) optimization parameters were standardized (AdamW optimizer, initial learning rate 0.0001, weight decay 0.01, batch size 2); and 3) training duration was fixed at 100 epochs for all models. Convergence behavior was monitored across all experimental conditions. All models converged stably between epochs 60–80 during training. Preliminary experiments showed that the model converged and entered a performance plateau after approximately 50 epochs; therefore, the number of training epochs was set to 100 to ensure stable convergence. The source code, trained models and the pre-processing scripts were access by the link on Github (https://github.com/JosephXing/breast-nodule-sam).

### 2.5 Evaluation metric

The Dice correlation coefficient and the Hausdorff distance were used as the segmentation evaluation metric. Dice coefficient was a region-based segmentation metric designed to assess the region overlap between the ground truth and segmentation results, which was defined as Equation 4:

(4) $$\begin{array}{c} DSC(G,S)=\frac{2|G\cap S|}{\left|G|+|S\right|} \end{array}$$

Hausdorff distance was a boundary distance-based segmentation metric designed to evaluate the ground truth and segmentation result boundary distance, which was defined as Equation 5:

(5) $$\begin{array}{c} HD(G,S)=\max(\underset{g\in G}{\max}\{\underset{s\in S}{\min}∥s-g∥\},\underset{s\in S}{\max}\{\underset{g\in G}{\min}∥g-s∥\}) \end{array}$$

where *G =* {*g_1_, g_2_, …, g_N_*}, *S =* {*s_1_, s_2_, …, s_N_*} were the ground truth and segmentation results, respectively. The 95% Hausdorff distance was used to filter out outliers.

As the segmentation metrics fail to satisfy the normal distribution assumption, we first perform the paired t-tests on the performance of various methods, followed by the Nemenyi *post-hoc* pairwise comparisons if the p-value < 0.05. Additionally, a paired t-test was first applied to evaluate the overall difference among all methods; the resulting P-value < 0.05 indicated significant global disagreement. Subsequently, pairwise comparisons were performed using the Nemenyi *post-hoc* test to identify which specific pairs of methods differ significantly. All statistical analysis was conducted with Python, and P<0.05 was considered statistically significant.

## 3 Results

**Figure 2:**
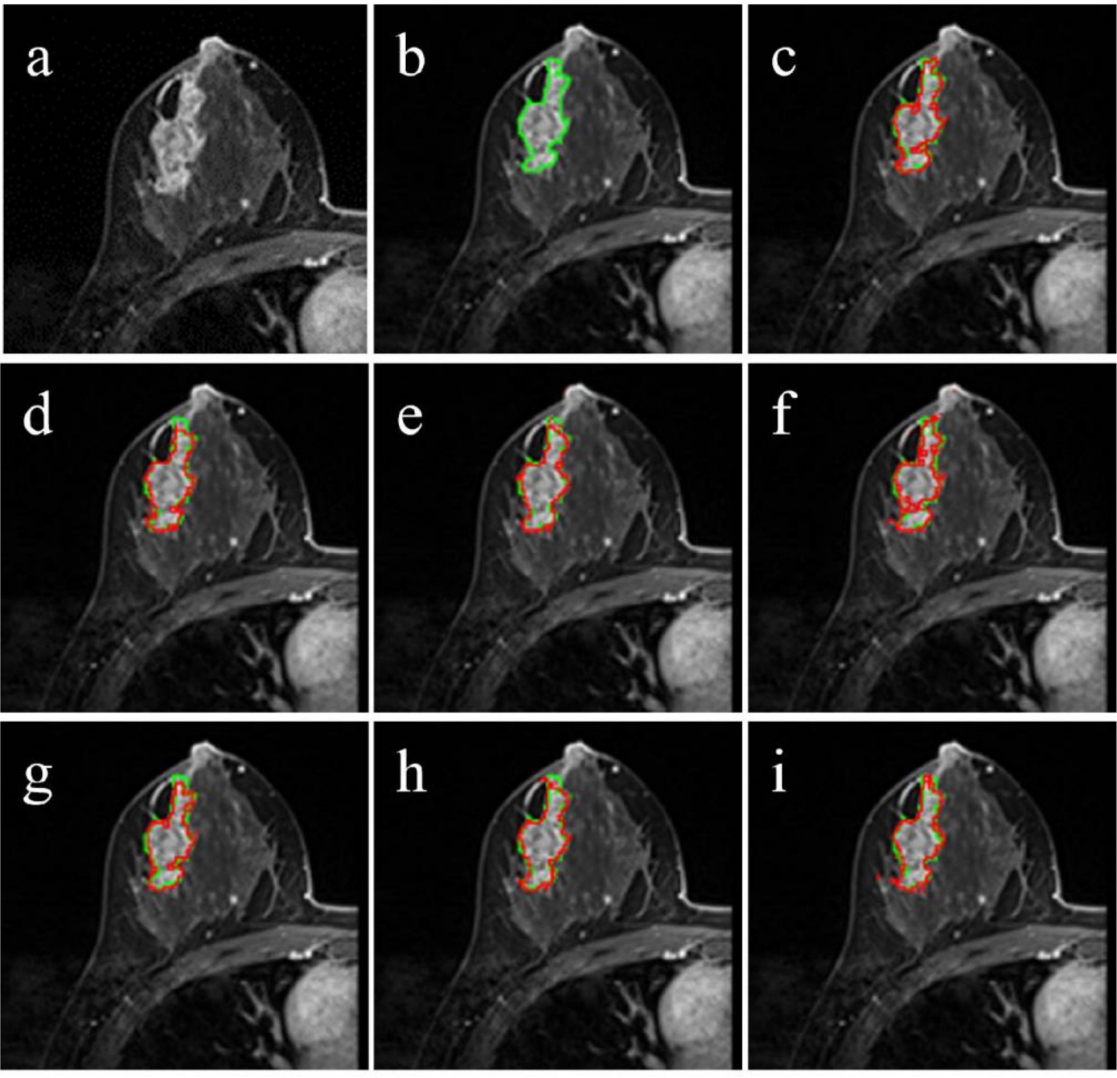
Segmentation results with good performance. The green line was ground truth boundary, and the red one was the segmentation result. **(a)** the original image, **(b)** the ground truth, **(c)** the SAM segmentation result, **(d)** MedSAM-m segmentation results, **(e)** MedSAM-a segmentation results, **(f)** MedSAM-nm segmentation results, **(g)** SAM-nm segmentation results, **(h)** MedSAM-anm segmentation results, **(i)** MedSAM segmentation results.

**Figure 3:**
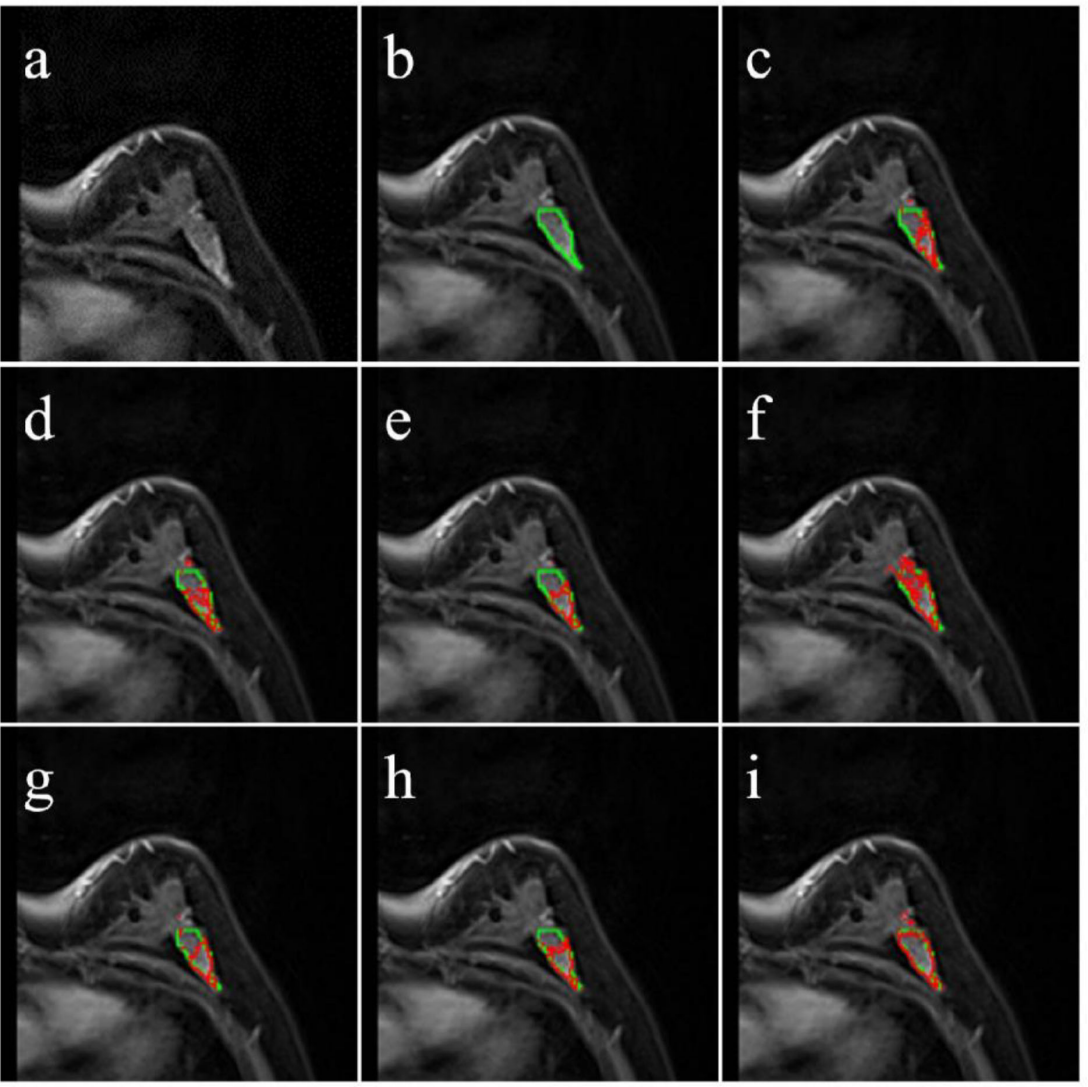
Segmentation results with poor performance. The green line was ground truth boundary, and the red one was the segmentation result. **(a)** the original image, **(b)** the ground truth, **(c)** the SAM segmentation result, **(d)** MedSAM-m segmentation results, **(e)** MedSAM-a segmentation results, **(f)** MedSAM-nm segmentation results, **(g)** SAM-nm segmentation results, **(h)** MedSAM-anm segmentation results, **(i)** MedSAM segmentation results.

**Figures 2** and **3** illustrated the segmentation results of different models, and the red boundary were the outline of the segmentation result, the green boundary was the ground truth. The segmentation boundary of MedSAM (**Figures 2**, **3** (i)) was clearer, smoother and more unambiguous than other segmentation results, and closer to the ground truth.

**Table 2** gave the average Dice of different models, and the overall segmentation performance of MedSAM was the best among all models, followed by MedSAM-anm, MedSAM-nm, SAM-nm, MedSAM-a, and SAM, MedSAM-m had the worst results.

**Table 2:** Dice values of different segmentation results.

Fold	Unet	Unetr	SAM	MedSAM-m	MedSAM-a	MedSAM-nm	SAM-nm	MedSAM-anm	MedSAM
Fold0	59.39 ± 34.08	55.34 ± 26.23	80.44 ± 12.16	79.03 ± 14.13	79.96 ± 13.58	80.73 ± 12.85	81.54 ± 10.53	80.04 ± 13.09	81.38 ± 19.00
Fold1	56.02 ± 28.98	55.79 ± 22.15	72.93 ± 19.00	72.39 ± 18.45	66.91 ± 19.96	75.21 ± 12.58	73.88 ± 14.78	76.86 ± 14.18	64.63 ± 23.39
Fold2	66.06 ± 25.32	60.50 ± 21.12	77.94 ± 11.85	76.80 ± 11.73	79.58 ± 10.08	78.82 ± 11.65	78.61 ± 11.84	81.63 ± 10.20	87.74 ± 12.30
Fold3	50.33 ± 31.06	55.84 ± 21.88	75.99 ± 14.08	74.02 ± 16.16	77.10 ± 10.08	76.67 ± 11.29	75.88 ± 10.53	80.41 ± 8.65	87.60 ± 9.14
Fold4	55.42 ± 30.80	60.10 ± 22.65	75.36 ± 15.28	73.61 ± 14.95	77.92 ± 14.96	78.06 ± 9.57	77.45 ± 15.32	80.67 ± 7.18	83.26 ± 13.17
All	57.46 ± 30.63	57.49 ± 22.99	76.54 ± 14.91	75.19 ± 15.44	76.28 ± 15.00	77.9 ± 11.82	77.47 ± 13.00	79.92 ± 11.13	80.90 ± 18.37

**Table 3** showed HD95 value of different models. MedSAM-nm segmentation results were the best, MedSAM-nm results were the second-best, followed by MedSAM, MedSAM-a, MedSAM-nm, and SAM.

**Table 3:** HD95 results of different segmentation.

Fold	Unet	Unetr	SAM	MedSAM-m	MedSAM-a	MedSAM-nm	SAM-nm	MedSAM-anm	MedSAM
Fold0	21.97 ± 24.22	68.68 ± 34.88	5.43 ± 5.97	6.97 ± 8.03	5.32 ± 4.10	5.16 ± 6.93	4.86 ± 5.95	5.37 ± 6.01	7.54 ± 8.22
Fold1	24.34 ± 20.95	44.09 ± 26.97	10.62 ± 8.30	10.20 ± 8.55	15.59 ± 13.63	8.77 ± 6.93	8.98 ± 7.00	7.44 ± 8.40	16.74 ± 10.60
Fold2	25.43 ± 26.78	52.29 ± 32.99	10.79 ± 16.49	8.12 ± 6.52	6.40 ± 5.37	6.52 ± 8.18	5.53 ± 6.02	3.47 ± 1.89	6.10 ± 9.24
Fold3	17.91 ± 20.23	67.03 ± 33.79	11.46 ± 18.77	18.30 ± 23.16	21.80 ± 31.45	6.43 ± 6.36	9.23 ± 10.41	3.50 ± 1.66	3.77 ± 4.11
Fold4	19.59 ± 24.85	67.55 ± 39.43	14.15 ± 29.57	8.49 ± 5.81	4.35 ± 3.13	5.85 ± 4.54	5.88 ± 6.38	3.30 ± 1.14	6.11 ± 6.62
All	21.87 ± 23.69	59.87 ± 35.23	10.45 ± 17.97	10.43 ± 12.96	10.76 ± 17.19	6.55 ± 6.82	6.91 ± 7.58	4.63 ± 5.06	8.07 ± 9.27

**Table 4** showed the nodule segmentation results for benign and malignant nodule cases. The segmentation results of malignant nodules were significantly higher than those of benign nodules. In the segmentation of malignant nodules, the Dice value of MedSAM segmentation remained best, but in the benign segmentation results, and the Dice value of MedSAM-nm was the best.

**Table 4:** Nodule segmentation results in malignant and benign nodule cases.

Tumor Type	Metric	SAM	MedSAM-m	MedSAM-a	MedSAM-nm	SAM-nm	MedSAM-anm	MedSAM
malignant	Dice	77.94 ± 13.93	76.53 ± 14.81	78.11 ± 12.44	78.91 ± 11.36	78.91 ± 11.36	80.94 ± 10.16	83.68 ± 15.75
	Hd95	9.44 ± 14.02	10.25 ± 13.40	7.34 ± 18.45	6.39 ± 3.71	6.28 ± 6.21	4.45 ± 4.79	7.34 ± 9.12
benign	Dice	70.68 ± 14.72	69.52 ± 16.70	68.56 ± 10.20	73.62 ± 7.25	71.41 ± 9.56	75.62 ± 13.70	69.17 ± 23.31
	Hd95	14.72 ± 28.86	11.22 ± 10.86	10.20 ± 10.28	7.25 ± 7.22	9.56 ± 11.32	5.39 ± 6.01	11.18 ± 9.24

To evaluate the model’s segmentation performance for small lesions, tests were also conducted on four rejected cases, with the results presented in **Table 5**. The metrics obtained of each model was relatively poor, far lower than those of the 99 large nodule samples.

**Table 5:** Segmentation results of 4 small nodules cases.

Metric	SAM	MedSAM-m	MedSAM-a	MedSAM-nm	SAM-nm	MedSAM-anm	MedSAM
Dice	42.53 ± 17.46	34.40 ± 16.88	38.14 ± 17.68	43.14 ± 17.55	44.25 ± 16.26	41.15 ± 22.89	29.64 ± 17.11
HD95	37.93 ± 22.68	27.99 ± 21.57	18.87 ± 14.83	27.51 ± 22.97	26.66 ± 23.71	22.89 ± 20.73	26.34 ± 17.32

## 4 Discussion

The purpose of this study was to explore the practical influence of various factors on the segmentation results of breast nodules using the SAM model and contributed to an evolving landscape of SAM adaptation methodologies for medical imaging. Three influencing factors, the initial weight, the breast mask and the prompt box were considered. The performance of seven models were compared with Dice and HD95. Our full fine-tuning of MedSAM weights achieved superior Dice performance on our limited dataset (n = 99).

Our investigation specifically addresses dynamic contrast-enhanced (DCE) MRI, a modality fundamentally distinct from mammography and ultrasound. DCE-MRI captures tumor vascular kinetics through temporal enhancement patterns, offering superior sensitivity for dense breast tissue and invasive lobular carcinomas compared to mammography’s structural assessment. However, this modality suffered from small lesion size, low contrast-to-noise ratios in non-enhanced phases, and complex parenchymal backgrounds. These factors may lead to suboptimal performance when direct transfer of SAM configurations was optimized for ultrasound or mammography (30–32). Additionally, our findings are modality-specific: the fixed prompt box strategy optimized in the present study assumes DCE-MRI`s typical lesion conspicuity patterns and may require recalibration for T2-weighted or diffusion-weighted sequences where tumor boundaries exhibit different intensity.

**Figure 4:**
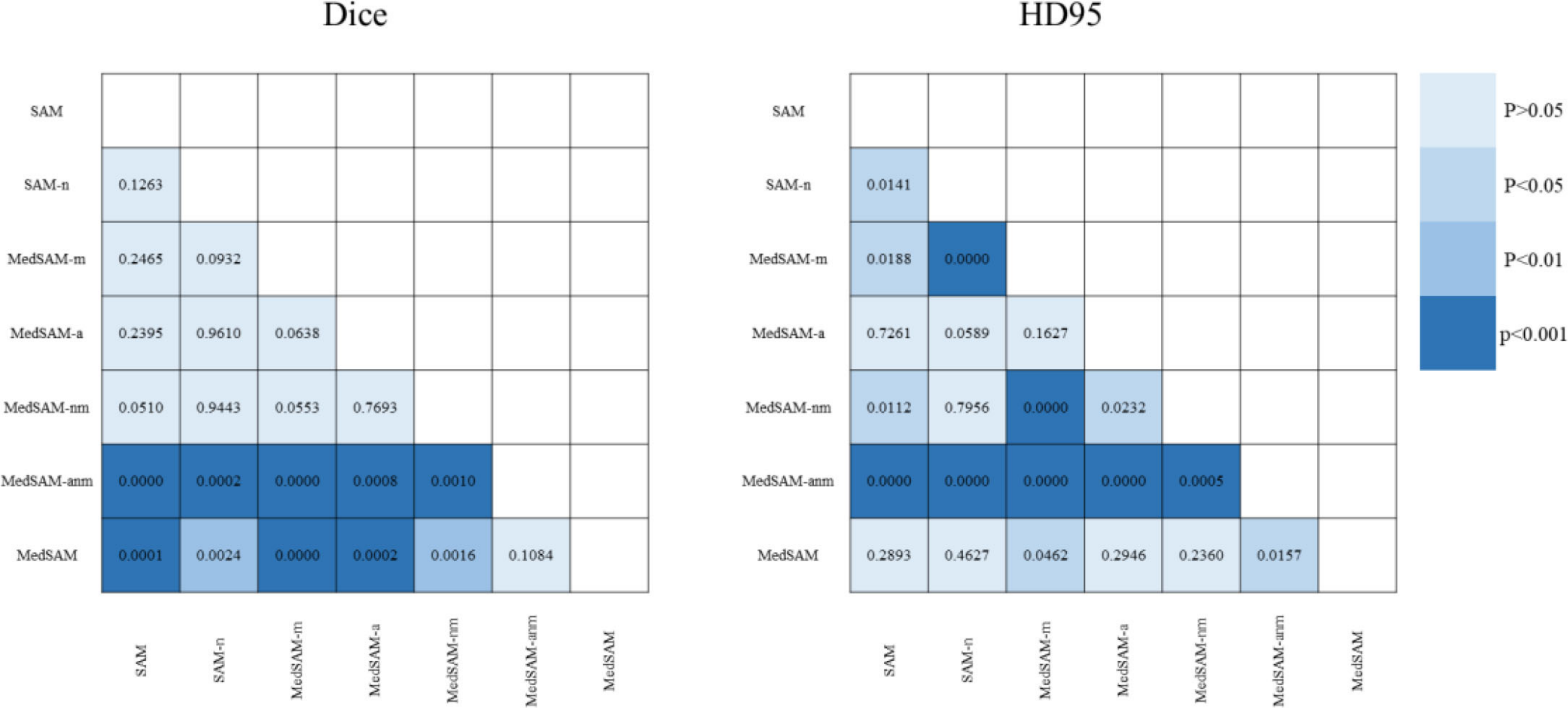
Nemenyi *post-hoc* pairwise comparisons, the left one is the Dice results, and the right is the HD95 results.

From the perspective of Dice alone, the average Dice of MedSAM was the largest, and the second best was MedSAM-anm.And the difference between the two was small, the Nemenyi test (**Figure 4**) indicated that there was a significant difference between the two. For HD95 index, the average result of MedSAM-anm was significantly smaller than that of MedSAM, and the P-value indicated a significant difference between the two. The above analysis showed that MedSAM-anm segmentation results were better. However, the prompt box used by MedSAM-anm was an adaptive prompt box, which required a very accurate nodule prompt box in advance, which was not feasible in practical applications, but is only an ideal state. In summary, the MedSAM segmentation method was superior.

Compared with MedSAM, the average index of SAM was worse in both Dice and HD95 indicators, and the p-values were 0.0003 and 0.2893, respectively, which were significantly different in Dice, indicating that the segmentation results were improved by training with MedSAM`s initial parameters. It was reflected that the addition of other medical images in the training model was conducive to the model to learn more and more accurate characteristics of breast nodules, and had a certain effect on the segmentation of breast nodules. The performance on small nodules (<50 voxels) stems from resolution loss, fixed box expansion and architectural limitation. The interpolation artifacts had a significant impact on small nodules. In addition, the architectures used in the present study were pretrained on natural images with high-frequency texture, lacks sensitivity to MRI’s subtle intensity gradients characteristic of early-stage lesions. All these factors contributed a related poor performance on small nodules.

MedSAM was better than MedSAM-a, but if the segmentation results of malignant and benign nodules were separated, MedSAM was better than MedSAM-a when dealing with the segmentation of benign nodules, which showed that adaptive box were more effective when splitting small targets, but adaptive box were not advisable in practical applications.

Multiplying the breast mask with input image, i.e., the MedSAM-m method, the segmentation results obtained were relatively poor, so it was not appropriate to add the breast mask in this way. The average values of MedSAM segmentation metric (Dice and HD95) were better than those of MedSAM-nm for the models with and without breast masks, but the Nemenyi tests of the two were P = 0.2465 and P = 0.0188, respectively. Considering that the breast mask was much larger than the nodular area, this can easily lead to some false positive areas, but it could still provide some effective information for segmentation to improve the segmentation result.

Architectural hybrids represent an alternative adaptation axis. SAM-UNet (33) and SAM-Med2.5D augment SAM’s encoder with custom decoders or multi-plane aggregation, achieving reported a high Dice. Our optimized MedSAM configuration (80.90% Dice) approaches but does not exceed these figures, likely reflecting the inherent difficulty of breast MRI segmentation rather than methodological inferiority. Crucially, our approach requires no architectural modification, offering deployment simplicity that may outweigh marginal performance gains in resource-limited settings. Pending multi-center prospective studies, we recommend integration of our proposed configuration with automated detection modules to realize fully automated pipelines. The broader potential of SAM in breast MR imaging remains to be fully elucidated.

There were some limitations in this paper. Firstly, the amount of data is relatively small, only 103 cases, and the benign and malignant data were uneven. In the tasks for small-scale, specialized applications like breast MRI, the SAM-based methods may be outweighed by the representational benefits of task-specific gradient updates. Secondly, the data used in this article are all single breast nodule data, lacking cases of multiple nodules. Although the results of nodule segmentation in this paper was impressive, they still do not have high results, which was consistent with the conclusions in (34). Finally, in most clinical scenarios, especially diagnosis and intervention, the two metrics are equally important: DICE has a slight advantage in nodule screening, while HD95 is slightly preferable in clinical operations. The ultimate goal of breast nodule segmentation algorithms is to obtain high values for both metrics simultaneously.

## 5 Conclusion

In this paper, we present a pragmatic, empirically-grounded investigation of SAM configuration for breast MRI nodule segmentation. For the practical application of SAM in breast nodule segmentation, different segmentation experiments were carried out on the initial weight, prompt box type and breast mask. The results demonstrate that MedSAM initialization combined with patient-specific fixed prompt boxes yields promising segmentation performance in a single-center cohort, while identifying the limited utility of anatomical masking. These findings provide actionable guidance for clinicians and researchers seeking to deploy foundation models in resource-constrained environments without extensive architectural engineering.

## Data Availability

The original contributions presented in the study are included in the article/supplementary material. Further inquiries can be directed to the corresponding authors.
